# Autoimmune Endocrine Dysfunctions Associated with Cancer Immunotherapies

**DOI:** 10.3390/ijms20102560

**Published:** 2019-05-24

**Authors:** Silvia Martina Ferrari, Poupak Fallahi, Giusy Elia, Francesca Ragusa, Ilaria Ruffilli, Armando Patrizio, Maria Rosaria Galdiero, Enke Baldini, Salvatore Ulisse, Gianni Marone, Alessandro Antonelli

**Affiliations:** 1 Department of Clinical and Experimental Medicine, University of Pisa, Via Savi 10, 56126 Pisa, Italy; sm.ferrari@int.med.unipi.it (S.M.F.); e.giusy_87@hotmail.it (G.E.); francescaragusa86@gmail.com (F.R.); ilaria.ruffilli@gmail.com (I.R.); armandopatrizio125@gmail.com (A.P.); 2 Department of Translational Research and New Technologies in Medicine and Surgery, University of Pisa, Via Savi 10, 56126 Pisa, Italy; poupak.fallahi@unipi.it; 3 Department of Translational Medical Sciences and Center for Basic and Clinical Immunology Research (CISI), University of Naples Federico II, 80131 Naples, Italy; mrgaldiero@libero.it (M.R.G.); marone@unina.it (G.M.); 4 WAO Center of Excellence, 80131 Naples, Italy; 5 Department of Experimental Medicine, ‘Sapienza’ University of Rome, 00161 Rome, Italy; enke.baldini@uniroma1.it (E.B.); salvatore.ulisse@uniroma1.it (S.U.); 6 Institute of Experimental Endocrinology and Oncology “Gaetano Salvatore” (IEOS), National Research Council (CNR), 80131 Naples, Italy

**Keywords:** PD-1, PD-L1, CTLA-4, immune checkpoint inhibitors, thyroid disorders, hypophysitis

## Abstract

Immune checkpoint inhibitors block the checkpoint molecules. Different types of cancer immune checkpoint inhibitors have been approved recently: CTLA-4 monoclonal antibodies (as ipilimumab); anti-PD-1 monoclonal antibodies (as pembrolizumab and nivolumab); and anti-PD-L1 monoclonal antibodies (as atezolizumab, avelumab, and durmalumab). We collect recent published results about autoimmune endocrine dysfunctions associated with cancer antibody immunotherapies. These agents cause a raised immune response leading to immune-related adverse events (irAEs), varying from mild to fatal, based on the organ system and severity. Immune-related endocrine toxicities are usually irreversible in 50% of cases, and include hypophysitis, thyroid dysfunctions, type 1 diabetes mellitus, and adrenal insufficiency. Anti-PD-1-antibodies are more frequently associated with thyroid dysfunctions (including painless thyroiditis, hypothyroidism, thyrotoxicosis, or thyroid storm), while the most frequent irAE related to anti-CTLA-4-antibodies is hypophysitis. The combination of anti-CTLA-4 and anti-PD-1 antibodies is associated with a 30% chance of irAEs. Symptoms and clinical signs vary depending on the target organ. IrAEs are usually managed by an oncological therapist, but in more challenging circumstances (i.e., for new onset insulin–dependent diabetes, hypoadrenalism, gonadal hormones dysfunctions, or durable hypophysitis) an endocrinologist is needed.

## 1. Introduction

Cancer immunotherapies unleash the immune system to control malignancy. The use of immunotherapy incorporated into adjuvant and neoadjuvant cancer therapies [1], a bispecific T–cell engager, immune checkpoint inhibitors (ICIs) [2,3], and Talimogene laherparepvec (T-VEC) (the first oncolytic immunotherapy) [4] have been recently approved.

Additionally, the importance of cytokines and chemokines and their possible modulation are still under evaluation in human cancer [5,6,7].

The immune system has the capability to recognize and destroy non-self or cancer cells: T cells recognize and interact with an antigen-class II major histocompatibility complex (MHC) on the membrane of the antigen-presenting cells (APC).

Traditional cancer therapies cause tumor cell death and the subsequent release of various new antigens, which are recognized as non-self in the lymph nodes that drain the tumor, thereby activating tumor immunity. Then, T cells enter the circulation reaching the tumor, infiltrate it, and induce malignant cell lysis with a further release of tumor antigens, reiterating the process [8].

Immune checkpoints are crucial for maintaining self–tolerance and regulating the immune system, preventing it from attacking cells in a random manner. Stimulatory checkpoint molecules are part of the tumor necrosis factor (TNF) receptor superfamily (cluster of differentiation (CD)27, CD40, GITR, CD137, and OX40), whereas CD28 and ICOS belong to the B7–CD28 superfamily; moreover, distinct inhibitory checkpoint molecules exist.

T cell–mediated inhibitory signaling pathways allow tumor growth to induce tolerance of the tumor antigens. The role of cytotoxic T lymphocyte antigen 4 (CTLA–4) as a molecular target for cancer immunotherapy was shown for the first time in 1996 [9]. Ever since then, different T cell receptors (programmed cell death protein–1 (PD–1), lymphocyte activation gene–3 (LAG–3), ì programmed death-ligand 1 (PD–L1), T–cell immunoglobulin mucin protein–3 (TIM–3), CD–137, and GITR) have been identified as possible targets to develop new therapeutics [10]. ICIs are able to block inhibitory checkpoint molecules, thus breaking the immune tolerance to tumor-associated antigens [11].

**Figure 1:**
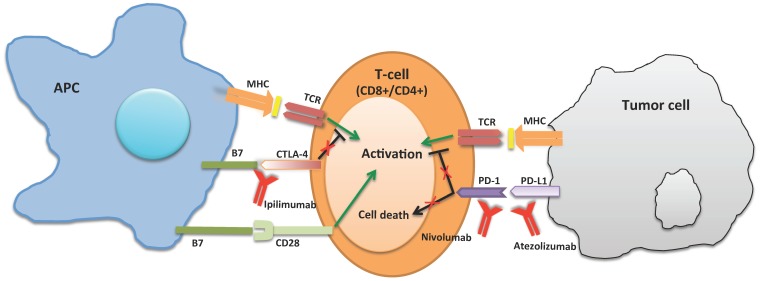
Anti CTLA-4 (such as Ipilimumab) increased the T-cell activation by binding the CTLA-4 receptor. Anti PD-1 (such as Nivolumab) and anti PD-L1 (such as Atezolizumab) allow the T-cell to identify the tumor cells binding to PD-1, or PD-L1, respectively.

CTLA–4 (or CD152) is a negative regulator of T cell activation, acting as an immune checkpoint. CTLA–4 is constitutively expressed in regulatory T cells and is upregulated in conventional T cells after the phenomenon of activation, which is considerable in cancer. Once bound to B7 (B7 has been distinguished in two subtypes, B7.1 or CD80, and B7.2 or CD86) on the surface of APC cells, it functions as an “off” switch (Figure 1). The evaluation of the possible use of antagonistic antibodies against CTLA–4 (as ipilimumab, the first approved immune checkpoint blockade drug) is ongoing, in order to inhibit the immune system tolerance to tumors, in this way possibly supplying a useful immunotherapy strategy for patients with cancer. Tremelimumab is another CTLA-4 inhibiting monoclonal antibody that it is not approved yet [12,13].

The cell surface receptor PD–1 (or CD279) is determinant in down–regulating the immune system and suppressing T–cell inflammatory activity, promoting self tolerance (Figure 1).

PD–1 promotes apoptosis (programmed cell death) of antigen–specific T–cells in lymph nodes and increases regulatory T-cell (antiflammatory, suppressive T cells) survival. In this way, autoimmune reactions are prevented, but, on the other hand, cancer cells can escape the killing from the immune system [14].

Several cancer immunotherapy agents targeting PD–1 have been developed.

One such anti–PD–1 antibody drug, nivolumab, produced complete or partial responses in non–small–cell lung cancer (NSCLC), melanoma, and renal–cell cancer in clinical trials and was approved to treat metastatic melanoma in July 2014 in Japan and in December 2014 by the US FDA [15,16].

Moreover, pembrolizumab, targeting PD–1, was approved to treat metastatic melanoma in September 2014 by the FDA. From 2015 until today, it has been approved by FDA for the treatment of NSCLC patients whose disease has progressed despite other treatments [17,18], such as for those with advanced melanoma (2015), recurrent or metastatic head and neck squamous cell carcinoma (2016), classical Hodgkin lymphoma (2017), as first-line combination therapy for patients with metastatic NSCLC, irrespective of PD-L1 expression (2017), locally advanced or metastatic urothelial carcinoma (2017), any solid tumor with a specific genetic feature (2017), for previously treated patients with recurrent locally advanced or metastatic gastric or gastroesophageal junction cancer whose tumors express PD-L1 (2017), for previously treated patients with recurrent or metastatic cervical cancer whose tumors express PD-L1 (2018), refractory or relapsed primary mediastinal large B-cell lymphoma (2018), metastatic nonsquamous NSCLC with no EGFR or ALK genomic tumor aberrations (2018), in combination with carboplatin and either paclitaxel or nab-paclitaxel for the first-line treatment of patients with metastatic squamous NSCLC (2018), for patients with hepatocellular carcinoma who have been previously treated with sorafenib (2018), recurrent locally advanced or metastatic Merkel cell carcinoma (2018), melanoma with involvement of lymph node(s) following complete resection (2019), or in combination with inlyta (axitinib) as a first-line treatment for patients with advanced renal cell carcinoma (2019) [19].

Pidilizumab (CT–011, Cure Tech) and BMS–936559 (Bristol Myers Squibb) are other drugs targeting PD–1 currently under investigation [20].

The 40 kDa type 1 transmembrane protein PD–L1 seems to have a determinant role in suppressing the immune system, for example, during autoimmune diseases and other disease states (as hepatitis), tissue allografts, and pregnancy. Generally, the immune system reacts against non-self-antigens associated with endogenous or exogenous danger signals, provoking the proliferation of CD4+ helper cells and/or antigen–specific CD8+ T cells [8,10,21]. Once PD–L1 is bound to PD–1 or B7.1, an inhibitory signal decreases the proliferation of antigen–specific T–cells in lymph nodes, also reducing the apoptosis rate of anti–inflammatory, suppressive T cells [21].

Upregulation of PD–L1 allows tumors to escape from the host immune surveillance; in fact, analyzing 196 tumor specimens of renal cell carcinoma, high PD–L1 expression was associated with increased tumor aggressiveness and a 4.5–fold increased risk of death.

Different clinical trials are ongoing to investigate PD–L1 inhibitors (as durvalumab, atezolizumab, and avelumab) as immuno–oncology therapies with promising results [22,23,24].

In this review, we collect published recent results about autoimmune endocrine dysfunctions associated with cancer immunotherapies, in particular those with immune checkpoint inhibitors. These agents cause a raised immune response leading to endocrine immune–related adverse events (irAEs) that vary from mild to fatal.

## 2. ICIs target Different Tumor Types

ICIs unleash the immune system to control cancer, regardless of cancer histology or the presence/absence of driver mutations. ICIs show different adverse effects compared to chemotherapy and targeted therapies, and their combination with other ICIs or other treatments improves the effectiveness of immunotherapy [8,25].

As an example, ipilimumab has durable effects in mucosal, ophthalmic, and cutaneous melanomas, which have distinct biology [26,27,28,29,30,31], and showed no differences in time of response or overall survival (OS) considering melanomas with NRAS and BRAF mutations [32].

The efficacy of anti-PD–1 antibody treatment has been shown in numerous kinds of tumors, including Hodgkin’s lymphoma, esophageal and gastric tumors, small–cell lung cancer (SCLC), NSCLC, kidney, hepatocellular, bladder, head and neck, and breast cancers [25].

Other ICIs are under investigation to treat solid tumors and haematological neoplasms [25], even if the situation may be different in the case of hematological malignancies [33,34,35].

## 3. ICIs-Associated Toxicities

Activating the immune system to eradicate cancer cells, ICIs can increase the risk of developing autoimmune diseases [36,37,38]. The medical literature reports many cases of immunotherapy–related autoimmune diseases that required the treatment interruption and/or the administration of glucocorticoids or other immunosuppressive drugs for their control [36,37,39]. Various types of autoimmune diseases ranging from organ–specific to systemic illnesses have been associated with immunotherapy [36,39,40,41] (Table 1).

Every organ (muscles, skin, bowels, lungs, heart, endocrine tissues, liver, kidneys, central nervous system, and eyes) can be damaged by ICIs, as they activate immune cells against self-antigens [42], even if the exact mechanisms of most irAEs have not been revealed yet. Clinical trials report the presence of anti–PD–1/PD–L1 antibody-associated grade 3–5 adverse events in approximately 7–19% of treated patients. ICIs are discontinued, owing to adverse events, with a rate of about 3–8% for anti–PD–1/PD–L1 antibodies, up to 15% for ipilimumab, and even higher (36%) for the combination of nivolumab and ipilimumab [8]. Toxicities are shown after months or years from the final dose of ICIs [42].

The most common side effects induced by treatment with ipilimumab involve skin (cutaneous rash and pruritus), the gastrointestinal tract (diarrhoea and colitis), the endocrine system (thyroid dysfunctions and hypophysitis), and the liver (autoimmune hepatitis). Immune–related arthritis, uveitis, myositis, and neuropathy also occur on occasion [1].

The incidence of irAEs has been investigated in oncologic patients treated with ipilimumab and tremelimumab (anti–CTLA–4 antibodies) [42]. All–grade irAEs had an incidence of 72% (95% confidence interval (CI), 65–79%), and a high–grade of 24% (95% CI, 18–30%). IrAEs included skin lesions (rash, pruritus, and vitiligo) and colitis. Less frequent irAEs were hypophysitis, hepatitis, and thyroiditis. Sarcoidosis, Guillain–Barré syndrome, uveitis, polymyalgia rheumatic/Horton, and immune–mediated cytopenia were rare. The risk of irAEs correlated with the dosage, with all–grade irAEs showing an incidence of 61% (95% CI, 56–66%) for ipilimumab 3 mg/kg and 79% (95% CI, 69–89%) for ipilimumab (10 mg/kg). Approximately 0.86% of patients died due to irAEs [43].

A recent paper reviewed incidences and kinetics of onset and resolution of immune–mediated “adverse events of specific interest” (AEOSI), with the approved PD–1 inhibitors nivolumab and pembrolizumab. The severity of AEOSI was mild to moderate (grade 1–2); the frequency of immune–mediated but also idiopathic grade 3–4 adverse drug reactions was ≤2% for any event term. The reported irAES were gastrointestinal, dermatological, endocrine, pulmonary, hepatic and renal toxicities. Although the time of onset was not predictable (the median range from 1 to 6 months) most of the events were reversible. With a systemic use of glucocorticoids, especially methylprednisolone or equivalents, most AEOSI were well manageable. Non–steroidal immunosuppressants could be considered for certain cases of refractory/recalcitrant, long–lasting immune toxicities [44,45,46].

## 4. Immune–Related Endocrine Toxicities

Endocrine irAEs include hypophysitis, thyroid dysfunctions, type 1 diabetes mellitus (T1DM), and adrenal insufficiency. Endocrine disorders are often irreversible [47], and studies report recovery of the pituitary–thyroid axis and pituitary–gonadal axis in up to 50–60% of patients [11,48,49], while only few cases of pituitary–adrenal axis healing have been described [11] (Table 2).

### 4.1. Hypophysitis

Hypophysitis is a rare illness [11], as its incidence is less than 1% in surgically treated pituitary lesions [65]. On the contrary, the ipilimumab–induced incidence of hypophysitis was 0–17%, as reported by the clinical data obtained from the studies on ICI–induced hypophysitis for CTLA–4 [48,66], while that for tremelimumab (phase I and randomized clinical trial results) was 0.4–5% [66,67]. For nivolumab or pembrolizumab, the incidence was relatively lower at <1% for both [2,3]. An incidence of 8.0–11.7% for the development of hypophysitis has been reported by international oncology centres that use these new drugs for the treatment of cancer [51,52,68].

Ipilimumab–related autoimmune hypophysitis is more frequent in men, even if its idiopathic counterpart (lymphocytic hypophysitis) is more common in women [48,51,69]. Older age and male gender represent risk factors for ipilimumab–induced hypophysitis in patients [70].

The incidence of hypophysitis increased with higher doses and changed depending on the use of adjuvant therapy [48]. It was 1.8–3.3% in patients treated with low doses of ipilimumab (<3 mg/kg) [71] and 4.9–17% with doses >3 mg/kg (up to 9 mg/kg) [50].

Similar elevated rates of hypophysitis (grade 3 or 4 toxicity) were described in patients treated with ipilimumab and an adjuvant therapy (including a prostate–specific antigen vaccine [72], prostate cancer cell vaccine [73], or bevacizumab [74]), leading to the hypothesis that the adjuvant therapy did not change the toxicity profile.

Furthermore, one paper showed that a combination of ipilimumab and nivolumab did not affect the incidence of hypophysitis with respect to monotherapy [75], while another study reported the opposite results [51].

The prevalence of hypophysitis, the course of the disease, and the obtained results after the treatment with ipilimumab were investigated in 154 patients with metastatic melanoma [70]. The Authors showed an ipilimumab–induced hypophysitis in a significantly older population (mean age: 68.2 ± 2.4 years vs. 59.9 ± 1.0; *p* = 0.005) and concluded that male gender and older age can be considered to be risk factors [70].

#### Clinical Manifestations of Hypophysitis

The autoimmune inflammation of the hypophysis generally induces structural changes and the enlargement of the glands leading to a headache, which is one of the first symptoms, and hormonal disturbance [76,77]. The measured change in pituitary size is about of 5 mm [43]. Symptoms such as anorexia, fatigue, diarrhoea, weakness, and nausea are unspecific and could be associated with pituitary dysfunction or nonendocrine–related adverse events, while visual symptoms are rare [43]. Other symptoms have been described as confusion, loss of libido, hallucination, polyuria, polydipsia, memory loss, erectile dysfunction, cold intolerance, insomnia, and dizziness [43,66,77,78]. The presence of unspecific symptoms, in particular hyponatremia, hypotension, or hypoglycaemia, points to the necessity of additional endocrine evaluations. Owing to the possible fatal nature of untreated hypoadrenalism, these patients should be immediately evaluated. The time to onset of endocrine adverse events is approximately 9 weeks (with a range of 5–36 weeks) after the beginning of the therapy [68,69]. A case of hypophysitis occurring 19 months after the first ipilimumab infusion has also been described [51]. Therefore, longer term monitoring should be evaluated.

Adrenocorticotropic hormone (ACTH) and/or thyroid–stimulating hormone (TSH) deficiencies are the most common manifestations, and anterior hypopituitarism is more prevalent than diabetes insipidus [49,51,79]. Elevated or low levels of prolactin have been reported [80], and hypogonadotropic hypogonadism and low levels of insulin–like growth factor 1 (IGF1) can also be present [49]. A male gender and older age are considered risk factors for ICIs–related hypophysitis [53].

It is important to cautiously evaluate the basal hormonal assessment at the beginning of immunotherapy and to carry out a questionnaire regarding suspicious symptoms for hypophysitis (hypoglycemia, headache, weakness, nausea, fatigue, hypotension) and measurements of glucose (before each cycle), TSH, free thyroxine (FT4), electrolytes, and morning cortisol (9 am), as hypopituitarism and cancer can have common symptoms and laboratory results. Pituitary magnetic resonance imaging (MRI) and a complete endocrine work–up (follicle–stimulating hormone/luteinizing hormone, estradiol/testosterone, IGF–1, prolactin, TSH, FT4, cortisol (9am), ACTH) should be carried out in case of compression symptoms (visual defects, headache) and/or clinically suspicious hypophysitis.

When morning cortisol is <250 nmol/L or random cortisol is <150 nmol/L with clinically suspicious adrenal insufficiency, a dynamic ACTH testing should be performed and replacement therapy with glucocorticoids should be administered.

An MRI is necessary to exclude the new occurrence of brain metastases and to assess the pituitary status, as pituitary morphology can vary during the course of the disease, from mild to moderate diffuse enlargement with homogenous or heterogeneous enhancement after contrast administration with stalk thickening at disease onset, to a subsequent atrophy of the gland and empty sella. A normal MRI does not exclude hypophysitis, and management should be done according to the clinical presentation and hormonal evaluation. The pituitary morphology sometimes changes before function or biochemical disturbances, and this could be resolved after 1–8 weeks of glucocorticoid treatment [53].

Hypophysitis can be managed especially by HRT and evaluation of ICIs discontinuation and/or high–dose (immunosuppressive) steroid therapy. Generally, immunotherapy may be continued in patients with grade 1 (mild) hypophysitis, while for the other grades of toxicities, the therapy should be stopped and high–dose systemic steroids (0.5–2 mg/kg/day of prednisolone or equivalent) should be administered, finally moving to a physiological replacement dose of hydrocortisone or prednisolone [54]. Once clinical improvement has been reached and toxicity is grade 1 or less, immunotherapy can resume, and appropriate HRT should be added. The European Society of Medical Oncology (ESMO) has recently published the regarding guidelines [54].

The thyrotroph axis and gonadotroph function may be regained, but it is uncommon for corticotroph function to be restored. Low levels of prolactin lead to a supposed lack of recovery function, with a positive predictive value of 85.7%, a negative predictive value of 57.1%, a specificity of 88.9%, sensitivity of 50%, and accuracy of 66.7% [55].

### 4.2. Thyroid Disorders and Their Management

Thyroid diseases and alterations (such as hypothyroidism, thyrotoxicosis, painless thyroiditis, or even thyroid storm [11]) are reported in 1–6% of patients treated with anti–CTLA–4–antibodies [48], representing the second most frequent kind of irAEs [11,49].

Primary hypothyroidism is established biochemically with high TSH associated to low FT4 or triiodothyronine (T3) levels, whereas central hypothyroidism due to adenohypophysis impairment (i.e., hypophysitis) is outlined by low to mid normal TSH levels and low FT4 [49].

In clinical trials where ipilimumab was tested, 7.6% (4.3–11.0%) of treated patients showed new cases of secondary hypothyroidism and 5.6% (5.2–5.9%) showed primary hypothyroidism [49,50,56,57,58,59,60,61,62,70], although many of these studies lack detailed descriptions of clinical and laboratory assessments of hypothyroidism.

One hundred and fifty-six patients with melanoma in treatment with ipilimumab showed (retrospectively) an 8% comprehensive incidence of hypophysitis and a 6% incidence of hypothyroidism/thyroiditis, whereas the number of new cases of primary hypoadrenalism was not relevant [51]. The association of ipilimumab and nivolumab caused new cases of thyroiditis or hypothyroidism and hypophysitis in 22% and 9% of patients, respectively [51].

More recently, a Systematic Review and Meta-Analysis has analyzed the incidence of adverse endocrine events provoked by anti-CTLA-4, anti–PD–1, or anti-PD–L1 agents. PD-1/PD-L1 inhibitors had a higher incidence of thyroid dysfunction, especially hypothyroidism (pembrolizumab, 8.5%; 95% CI, 7.5–9.7; nivolumab, 8.0%; 95% CI, 6.4–9.8; ipilimumab, 3.8%; 95% CI, 2.6–5.5; PD-L1, 5.5%; 95% CI, 4.4–6.8) [81].

Euthyroid Graves’ Ophthalmopathy and other rare endocrine side effects were reported, too [82]. For example, 3 patients affected by metastatic melanoma begin to suffer of thyroiditis and euthyroid Graves’ Ophthalmopathy under ipilimumab with/without bevacizumab, therapy [82].

PD–1 antibodies caused at least one irAE in 39.0–54.2% of patients prescribed with it [49]. The most frequent event was hypothyroidism (about 5.9% of incidence), while hyperthyroidism was described in only 1.0–4.7% of patients [49].

After anti–PD–1 therapy for metastatic malignancies, 10 patients developed painless thyroiditis, 6 patients developed temporary thyrotoxicosis, while four patients showed anti–thyroid antibodies in the serum. Patients with thyrotoxicosis were managed with beta–blockers, with a subsequent frank remission of the disease, and then hypothyroidism. The patients became anti–thyroid antibody positive and managed with a thyroid HRT for a period of six months [83].

A further study reported a serological exacerbation of autoimmune thyroid disease in two patients in treatment with nivolumab, one affected by Hashimoto’s thyroiditis and one by subclinical Hashimoto’s thyroiditis [84].

More recently, the safety and effectiveness of anti-PD-1 antibodies in patients with autoimmune or inflammatory disorders have been analyzed [85]. Forty-five/53 patients with autoimmune or inflammatory disorders in the REISAMIC registry were identified. The cancer diagnoses included melanoma, NSCLC, and others. The most frequent pre-existing autoimmune or inflammatory disorders were psoriasis, vitiligo, Sjögren syndrome, rheumatoid arthritis, and thyroiditis. In patients treated with anti-PD-1 antibodies, pre-existing autoimmune or inflammatory disorders was associated with a significantly increased risk of irAEs [85].

A 61–year–old developed painless thyroiditis despite a negative history for thyroid disorders [86] after nivolumab therapy for a progressive NSCLC resistant to radiotherapy and chemotherapy [87]. After three administrations, bilateral eyelid ptosis and conjunctival redness with chemosis were present. Severe proptosis and complete ophthalmoplegia, associated with normal thyroid hormone levels and negative anti–thyroperoxidase or anti–TSH receptor antibodies, were reported. The computed tomography (CT) scan of the orbits was consistent with evident bilateral proptosis, with the expansion of the orbital adipose tissue and no thickened extraocular muscles. The inflammation of the periorbital adipose tissue was shown by a T2–weighted MRI. Nivolumab was withheld, and the patient was administered with a weekly dose of methylprednisolone intravenously for three cycles, attaining a notable improvement of the remaining chemosis. Although his thyroid function remained normal with no sign of autoimmunity, the patient succumbed due to massive hemoptysis one week later [87].

Even a case of symptomatic hypothyroidism-associated myositis, confirmed by histology, after nivolumab therapy, has been reported [88].

The association of nivolumab with ipilimumab had the most elevated incidence of hyperthyroidism (10%) and hypothyroidism (17%) of any grade [63,89].

In KEYNOTE–001 (NCT01295827), 51 patients affected by advanced NSCLC received pembrolizumab [64], and their thyroidal function and anti–thyroid antibody serum levels were checked in a prospective manner at each study visit, even prior to the first dose of therapy. At baseline only 3/51 patients were hypothyroid. Ten/48 patients needed HRT due to new onset hypothyroidism. Thyroid dysfunction was shown by 8/10 patients with anti–thyroid antibodies (80% vs. 8%, *p* < 0.0001). Thyroid dysfunction developed early during the treatment (median: 42 days), and 6/10 patients faced a temporary hyperthyroidism (before hypothyroidism) with subsequent remission. Hyperthyroidism and hypothyroidism had no clinical impact. Of note, pembrolizumab therapy was associated with a more elevated OS in those who had shown thyroid dysfunction (hazard ratio, 0.29; 95% CI 0.09–0.94; *p* = 0.04) [64].

Interestingly, another study noticed that a new onset of serum thyroglobulin antibodies (AbTg) was associated with prolonged survival [90]. AbTg has been measured in patients affected by colon (*n* = 8), prostate (*n* = 35), or pancreatic (*n* = 53) cancer, before and after the GVAX therapy (a cancer vaccine made by whole tumor cells genetically modified to release the immune stimulatory cytokine, granulocyte–macrophage colony–stimulating factor (GM–CSF), and irradiated to prevent cell division) alone (*n* = 34), associated with ipilimumab (*n* = 42), or before and after ipilimumab alone (*n* = 20). After GVAX, AbTg became detectable, regardless the histotype of the tumor (colon cancer 75%, pancreatic cancer 76% and prostate cancer 81%) and the combination with ipilimumab (75% in the case of GVAX alone, and 78% when coadministered with ipilimumab). Moreover, AbTg seroconversion was associated with relevant prolonged survival (*p* = 0.01 in pancreas and *p* = 0.005 in prostate cancer) [90].

In a further study, 177 patients affected by metastatic melanoma were evaluated prospectively after treatment with ipilimumab (*n* = 15), anti–PD–1 (nivolumab, pembrolizumab) (*n* = 103), or combined ipilimumab and anti–PD–1 (*n* = 59) [91], and the development of irAEs was investigated. Eighteen% developed an endocrine side effect (thyroid dysfunction, 14%; hypophysitis, 6%; autoimmune diabetes, 0.6%). Combined immunotherapy showed a higher incidence of single or multiple endocrinopathies with respect to anti–PD–1 alone (27% vs. 9% and 7% vs. 0% respectively, *p* < 0.01) [91].

Recently, the Society for Immunotherapy of Cancer (SITC) has instituted a multidisciplinary Toxicity Management Working Group, with the aim to release consensus guidelines on the evaluation and the management of ICIs-associated toxicities [92]. The American Society of Clinical Oncology (ASCO) and the National Comprehensive Cancer Network (NCCN) then issued clinical practice guidelines to manage ICIs toxicities [93,94].

Thyroid dysfunctions usually have not sign and/or symptoms and are evident only by routine biochemical tests, but sometimes patients with new onset thyroiditis complain of a sore throat, tachycardia and palpitations, and other symptoms of hyperthyroidism [95].

IrAEs are usually managed by the oncological therapist, but in more challenging circumstances (i.e., for new onset insulin–dependent diabetes, adrenal insufficiency, gonadal hormone dysfunctions, or hypophysitis) an endocrinologist is needed [95].

Validated protocols can help clinicians to make management decisions on ICIs–related endocrinopathies, but experience in this new field plays a crucial role. In front of asymptomatic TSH abnormal values, either higher the normal range, or lower, FT4 serum levels should be measured. In the case of hypothyroidism, to rule out concomitant hypoadrenalism, it is mandatory to check serum cortisol levels too, before starting thyroid HRT. Levothyroxine is usually initiated at a low dose (i.e., 50 mcg) and then adjusted as needed [95].

Before starting with ICIs therapy, thyroidal function should be evaluated and then reassessed every 8 weeks throughout the treatment period [8,96]. Grade 1–2 hyperthyroidism does not need ICIs withdrawal, but in case of grade 3 hyperthyroidism, the therapy should be interrupted and oral prednisolone (1–2 mg/kg/day) begun [97,98]. If needed, anti–thyroid therapies (as methimazole, carbimazole, or propilthyouracil) could be recommended. With severe hyperthyroidism of grade 4, ICIs should be withheld, and methylprednisone (1–2 mg/kg/day; IV; for 3 days) followed by oral prednisolone (1–2 mg/kg/day) should be used [97,99].

Early detection of sympotmatic hypothyroidism is mandatory to decide when to withhold ICIs [11,97,100].

### 4.3. T1DM

Patients treated with anti–PD–1 or –PD–L1 therapies can present ICIs–induced diabetes [101], as reported in different papers in the literature.

ICIs–induced insulin–dependent diabetes is not common but could be a clinically significant event. After the first report of ICIs–induced diabetes [102], more than 39 cases have been described in 22 different papers [103,104,105,106,107,108,109,110,111,112,113,114]. The most common type of cancer was melanoma, and the most frequently used ICI were PD–1 or PD–L1 mAbs. Diabetic ketoacidosis (DKA) was present in 81% of cases, revealing the severe nature of this adverse event and thyroid disease in 28%. HLA–DR4 was present in 40% (8/20) of the patients and a higher than expected rate of anti–GAD65 antibodies (47%) (12.7%) were reported, compared to the general population (12.7%) [101].

In a series from two academic institutions, among those treated with ICIs, the overall incidence of this form of diabetes was 0.9% during the 6 years period examined [101].

Although many biochemical and clinical features of this type of diabetes are similar to those of sporadic T1DM, the median age at the time of the onset is certainly higher at around 66 years, whereas it takes an average period of 6.2 weeks from the beginning of the ICIs therapy to emerge, even if a wide variability (1–52 weeks) was found.

The quick progression from normoglycemia to hyperglycemia is caused by the rapid loss of beta-cells; random C–peptide levels were not detectable or low in 88% at the initial onset of hyperglycemia [115]. At diagnosis, A1C levels are similar to those in patients with new onset T1DM.

According to the first study, 47% (18/38) of subjects showed anti–GAD65 similar to primary T1DM [101], even if these and other autoantibodies are detectable before the clinical onset in more than 95% of patients with T1DM [116].

A notable predominance of HLA–DR4–positive cases was shown by HLA typing, while DR3, DQ2, and DQ8 were not overrepresented. The frequency of the HLA–DR4 genotypes was higher than in the background population but also higher than in patients with T1DM, in which 42% were reported to be positive for any of the DR4 alleles (×2 test, *p* < 0.001) [101].

To date, ICIs–induced diabetes has tended to be permanent, whereas patients with thyroiditis can attain spontaneous remission. Even several attempts with glucocorticoid administration with high (50 mg/day) and low (20 mg/day) doses of prednisone showed no recovery from diabetes [107,117].

### 4.4. Adrenalitis

Adrenalitis and primary adrenal insufficiency linked to the treatment with ipilimumab have been described, even if only occasionally [117,118]. After four doses of ipilimumab, a woman with metastatic melanoma complained about a headache and fatigue; the serum analysis showed low morning cortisol and corticotropin levels associated with pituitary enlargement at the brain MRI scan. Therefore, a diagnosis of hypophysitis and secondary adrenal insufficiency was established and hydrocortisone was started. Later, during the follow-up, adrenal glands enlarged bilaterally but were of normal size before the therapy. Cosyntropin stimulation had no effect, indicating primary adrenal insufficiency. Then, adrenal glands turned back to a normal size after 6 weeks, suggesting an ipilimumab–induced autoimmune adrenalitis [117].

In a patient aged 79 treated with ipilimumab, new enlarged, bilateral, hypermetabolic adrenal glands have been described by radiological studies, suggesting the presence of an adrenalitis induced by the therapy, and not metastatic disease [118].

Among 256 patients administered with ipilimumab, two cases of primary adrenal insufficiency (0.8%) were described [49,117,118], while another paper reported a patient with hyponatremia associated nivolumab–related primary adrenal failure [119]. The patient’s fluorodeoxyglucose (FDG) positron emission tomography (PET) CT scan showed a bilaterally increased FDG activity in the adrenals, in agreement with the diagnosis of autoimmune adrenalitis [119]. Patients administered with nivolumab or tremelimumab, or a combination of anti–PD–1 and anti–CTLA–4 agents, showed adrenal insufficiency of unknown origin [120]. The majority of the studies do not report the etiology of adrenal insufficiency. Generally, the relative risk of adrenal insufficiency of any cause was significantly high (3.87; 95% CI, 1.12–13.41), as reported also by Abdel–Rahman et al. [121].

Hyponatremia could be caused by ACTH deficiency but also by primary adrenal failure, and, for this reason, the measurement of aldosterone, ACTH, and renin should be performed. A subclinical form of adrenalitis has also been reported after treatment with ICIs through radiological evidence of adrenalitis (with normal endocrine functions) [118]. In case of adrenal enlargement after the treatment with ICIs, the adrenal function should be evaluated by measuring ACTH and cortisol levels, and a Synacthen stimulation test should be done, in order to exclude primary adrenal failure.

## 5. Conclusions

The immune system can distinguish and kill the tumor cells through T–cells able to recognize cancer antigens as non-self. Traditional oncological therapies, such as chemotherapy and radiotherapy, lead to cancer cells death and the subsequent release of tumor antigens, which are then presented by dendritic cells in the tumor–draining lymph nodes in order to activate tumor immunity. As a result, tumor–specific T cells infiltrate the malignant mass, induce further cancer cells lysis and increase the releasing of tumor antigens, reiterating the process [8]. During their evolution, tumors can suppress the immune response enhancing immune checkpoint inhibitory activity.

ICIs block the inhibitory checkpoint molecules, and various types of them have been recently approved: ipilimumab (anti-CTLA-4 monoclonal antibodies); pembrolizumab and nivolumab (anti-PD-1 monoclonal antibodies); atezolizumab, avelumab, and durmalumab (anti-PD-L1 monoclonal antibodies).

These agents cause a raised immune response, leading to irAEs that vary from mild to fatal, based on the organ system and severity [122]. Immune-related endocrine toxicities include hypophysitis, thyroid dysfunctions, type 1 diabetes mellitus, and adrenal insufficiency, with studies showing pituitary–thyroid and pituitary–gonadal axis recovery in up to 50–60% of patients.

IrAEs onset uses to be within 7 to 10 weeks from the beginning of ipilimumab and nivolumab treatment, respectively [123].

About 0–29% of patients show endocrine irAEs [124]. PD–1/PD–L1 inhibitors and anti–CTLA–4 antibodies have distinct mechanisms of action, as the PD–1/PD–L1 pathway regulates inflammatory reactions in tumor microenvironments and peripheral tissues, and its activation occurs later during the immune response, while CTLA–4 is induced in T cells earlier in response to antigens [124]. For this reason, the incidence of the induced endocrinopathies is different.

The most common irAE of grade 3/4 induced by ipilimumab is hypophysitis; hypothyroidism and hyperthyroidism follow in order of frequency. Tremelimumab is associated with a few reported endocrinopathies (0–8.3%) [124].

The incidence of thyroid disorders (painless thyroiditis, hypothyroidism, thyrotoxicosis, or thyroid storm [11]) is about 10% in patients receiving anti–PD–1/PD–L1 alone [8,89,125], but euthyroid Graves’ ophthalmopathy and other rare endocrine diseases have also been reported [82].

The appearance of anti-thyroid antibodies, thyroiditis, or thyroid dysfunctions in patients administered with ICIs has been associated with a prolonged survival [90], suggesting they might be possible markers of a more potent immune activation [38].

Consensus guidelines for the evaluation and management of ICIs-associated irAEs have been recently released [92,93,94].

Symptoms and clinical signs vary depending on the target organ. IrAEs are usually managed by the oncological therapist, but in more challenging circumstances (i.e., for new onset insulin–dependent diabetes, adrenal insufficiency, gonadal hormones dysfunctions, or hypophysitis), an endocrinologist is needed [95]. Further studies are needed to investigate the mechanism by which ICIs induce IrAEs, to prevent and to treat them.

## Figures and Tables

**Table 1:** List of endocrine adverse events and recommendations.

		Hypophysitis	Hypothyroidism	Hyperthyroidism	Adrenalitis
Grade 1	Clinical symptoms	✓ Asymptomatic or mild symptoms	✓ Asymptomatic or mild symptoms	✓ Absence of symptoms	✓ Absence of symptoms
	Clinical management strategies	✓ Intervention not indicated	✓ Intervention not indicated	✓ Intervention not indicated	✓ Intervention not indicated
Grade 2	Clinical symptoms	✓ Mild symptoms such as headache, mood changes and fatigue ✓ Depending on age, mild impairment of the Instrumental Activities of Daily Living	✓ Symptomatic	✓ Symptomatic	✓ Moderate symptoms
	Clinical management strategies	✓ Minimal, local or noninvasive intervention indicated	✓ Mild impairment of the Instrumental Activities of Daily Living ✓ Thyroid replacement indicated	✓ It’s recommended thyroid suppression therapy ✓ Limiting instrumental ADL	✓ Medical intervention indicated
Grade 3	Clinical symptoms	✓ The disability can limit self care ✓ Severe or medically significant but not immediately life-threatening	✓ Self care limitation that affects the Activities of Daily Living ✓ Severe symptoms	✓ Self care limitation that affects the Activities of Daily Living ✓ Severe symptoms	✓ Severe symptoms
	Clinical management strategies	✓ Hospitalization or prolongation of existing hospitalization indicated	✓ Hospitalization recommended	✓ Hospitalization recommended	✓ Hospitalization recommended
Grade 4	Clinical symptoms	✓ Life-threatening consequences	✓ Life-threatening consequences	✓ Life-threatening consequences	✓ Life-threatening consequences
	Clinical management strategies	✓ Urgent intervention recommended	✓ Urgent intervention recommended	✓ Urgent intervention recommended	✓ Urgent intervention recommended
Grade 5		✓ Death	✓ Death	✓ Death	✓ Death

The National Cancer Institute has recommended that adverse events on patients with cancer chemotherapy be graded as per the Common Terminology Criteria for Adverse Events (CTCAE) Version 5.0.

**Table 2:** Immune–related endocrine toxicities.

	Anti-CTLA-4 (Prevalence of the Disease: %)	Refs.	Anti PD-1/Anti PD-L1 (Prevalence of the Disease: %)	Refs.	Combination (Prevalence of the Disease: %)	Refs.
**Hypophysitis**	0–17 with Ipilimumab	[44,47]	<1	[2,3]	not increased in comparison to monotherapy results	[50]
	0.4–5 with Tremelimumab	[47,48]				
**Hypothyroidism**	4.3–11.0 secondary hypothyroidism	[45,51,52]	5.9	[45]	22	[49]
	5.2–5.9 primary hypothyroidism	[53,54,55,56,57,58,59,60,61,62]			17 of any grade	[63,64]
**Hyperthyroidism**	2	[38]	1.0–4.7	[45]	10 of any grade	[63,64]
**Diabetes**	0	[38]	0–1	[38]	NR	[38]
**Adrenalitis**	<2	[38]	<2	[38]	<2	[38]
